# Association Between Anaerobic Coverage and Clinical Outcomes in Patients with Lung Abscess: A Nationwide Database Study in Japan

**DOI:** 10.3390/pathogens15101013

**Published:** 2026-09-24

**Authors:** Hiroyuki Matsumoto, Haruka Kanao, Hisayuki Shuto, Kazufumi Hiramatsu, Kiyohide Fushimi, Kosaku Komiya

**Affiliations:** 1 Respiratory Medicine and Infectious Diseases, Faculty of Medicine, Oita University, 1-1 Idaigaoka, Hasama-machi, Yufu 879-5593, Oita, Japan; hmatsumoto@oita-u.ac.jp (H.M.);; 2 Department of Medical Safety Management, Faculty of Medicine, Oita University, 1-1 Idaigaoka, Hasama-machi, Yufu 879-5593, Oita, Japan; 3 Department of Health Policy and Informatics, Institute of Science Tokyo, Graduate School of Medical and Dental Sciences, S1560/S1568 M&D Tower, 1-5-45 Yushima, Bunkyo-ku 113-8519, Tokyo, Japan

**Keywords:** lung abscess, anaerobic coverage, antimicrobial therapy, antimicrobial stewardship, nationwide database

## Abstract

Empirical antimicrobial therapy for lung abscess commonly includes anaerobic coverage, although evidence supporting its clinical benefit remains limited. We investigated the association between the extent of anaerobic coverage in initial therapy and clinical outcomes using the Japanese Diagnosis Procedure Combination database. Adults hospitalized between April 2022 and March 2023 with lung abscess (ICD-10 J85.2) were classified according to whether the initial antimicrobial regimen provided limited or extended anaerobic coverage and was continued for at least 3 consecutive days. Among 2800 patients, 161 received limited coverage, and 2639 received extended coverage. After propensity score matching, 161 patients in each group were compared. There were no significant differences in 30-day in-hospital mortality (6.2% vs. 6.2%), overall in-hospital mortality (12.4% vs. 10.6%), or length of hospital stay. Findings were broadly consistent across inverse probability of treatment weighting analyses and a sensitivity analysis using an alternative exposure definition based on the antimicrobial regimen administered on hospital day 1. We found no statistically significant evidence that extended anaerobic coverage was associated with improved clinical outcomes, although clinically important differences cannot be excluded.

## 1. Introduction

Antimicrobial resistance (AMR) is one of the most important public health challenges worldwide. The report by O’Neill estimated that, if current trends in antimicrobial resistance continue, deaths attributable to drug-resistant infections could reach approximately 10 million annually worldwide by 2050 [1]. The Global Burden of Disease study published in 2022 reported that approximately 1.27 million deaths were directly attributable to bacterial AMR in 2019, and that AMR was associated with approximately 4.95 million deaths [2]. Against this background, antimicrobial stewardship has been increasingly emphasized worldwide to optimize antimicrobial use, reduce unnecessary or inappropriate antimicrobial therapy, and minimize unintended consequences, including antimicrobial resistance and antimicrobial-associated adverse events [3].

Lung abscess is a microbial infection characterized by liquefactive necrosis of lung tissue and cavity formation. It commonly develops following aspiration of oral or upper airway secretions. Anaerobic bacteria residing in the oral cavity and upper airway have been considered major causative pathogens. Because of the presumed involvement of anaerobic bacteria, antimicrobial agents with activity against anaerobes, such as clindamycin and beta-lactam/beta-lactamase inhibitor combinations, have been widely used as empirical therapy for lung abscess [4]. In recent years, however, the need for routine anaerobic coverage in aspiration-related pulmonary infections has been re-evaluated. A systematic review in the Japanese Respiratory Society Guidelines for the Management of Pneumonia in Adults 2024 found no clear clinical benefit of routinely adding antimicrobial agents targeting anaerobes for aspiration pneumonia [5,6]. Similarly, the American Thoracic Society and Infectious Diseases Society of America guidelines for community-acquired pneumonia suggest not routinely adding anaerobic coverage for aspiration pneumonia unless lung abscess or empyema is suspected [7].

Lung abscess often develops following aspiration and has traditionally been considered an infection in which anaerobes are major pathogens. However, few studies have directly evaluated whether extended anaerobic coverage in initial treatment improves clinical outcomes in patients with lung abscess. Therefore, current treatment strategies may partly rely on microbiological assumptions and historical clinical experience, and the clinical significance of anaerobic coverage should be re-examined from the perspectives of AMR control and antimicrobial stewardship. Because lung abscess is a relatively uncommon disease, even multicenter studies may have difficulty enrolling a sufficient number of patients. Analysis using the Diagnosis Procedure Combination (DPC) database, a nationwide large-scale database in Japan, may therefore be useful [8]. Accordingly, this study investigated the association between anaerobic coverage in initial antimicrobial therapy and clinical outcomes in patients with lung abscess using the DPC database.

## 2. Materials and Methods

### 2.1. Data Source

This study was conducted using the nationwide DPC inpatient database. In Japan, more than 1600 acute care hospitals participate in the DPC system, and the database covers a large proportion of acute care hospitalizations [8]. The database includes information on age, sex, body mass index (BMI), activities of daily living (ADL) assessed using the Barthel Index, Hugh–Jones classification, comorbidities, smoking history assessed using the Brinkman Index, level of consciousness on admission assessed using the Japan Coma Scale (JCS), principal diagnoses, diagnosis prompting admission, complications, prescription data, length of hospital stay, intensive care unit (ICU) admission, and discharge outcomes. Diagnostic information is recorded using International Classification of Diseases, 10th Revision (ICD-10) codes and corresponding Japanese disease names [8,9].

This study was approved by the Ethics Committee of the Faculty of Medicine, Oita University (approval number: 3294; approval date: 25 November 2025). Because this was a retrospective observational study using existing data, the requirement for informed consent from individual patients was waived by the ethics committee.

### 2.2. Patients

Patients admitted for treatment of lung abscess to acute care hospitals participating in the DPC system between April 2022 and March 2023 were eligible for inclusion. From the DPC database, we extracted patients aged 18 years or older who had ICD-10 code J85.2 (lung abscess) recorded as the diagnosis that prompted admission. Because the DPC database does not include diagnostic evidence such as imaging findings or microbiological test results, the diagnosis of lung abscess was based on clinical diagnoses made at each participating institution.

The exclusion criteria were as follows: (1) second or subsequent hospitalizations during the study period, (2) no intravenous administration of a study antimicrobial agent on hospital day 1, and (3) death or discharge on hospital day 1. Patients remaining after these three exclusions were included in the sensitivity analysis based on antimicrobial exposure on hospital day 1, irrespective of subsequent treatment modification. For the primary analysis, we additionally excluded patients whose initial antimicrobial regimen was not continued for at least 3 consecutive days.

The study antimicrobial agents were ceftriaxone, cefotaxime, cefepime, cefozopran, levofloxacin, ampicillin–sulbactam, piperacillin–tazobactam, meropenem, doripenem, lascufloxacin, clindamycin, and metronidazole.

### 2.3. Data Collection and Outcomes

Baseline patient characteristics included age, sex, BMI, Barthel Index, Hugh–Jones classification, comorbidities, smoking history, and level of consciousness on admission. Comorbidities were assessed using the Charlson Comorbidity Index (CCI) [10]. We also collected information on oxygen therapy, mechanical ventilation, corticosteroid use, immunosuppressant use, catecholamine use, and ICU admission on hospital day 1.

Anaerobic bacteria include *Bacteroides* spp., *Prevotella* spp., *Fusobacterium* spp., and *Peptostreptococcus* spp. Because few antimicrobial agents completely lack activity against anaerobic bacteria, antimicrobial regimens were classified as providing either limited anaerobic coverage (LAC) or extended anaerobic coverage (EAC) according to a previously published classification and the reported antimicrobial spectra of individual agents [11,12]. Consistent with the conceptual framework used in a previous study of aspiration pneumonia [11], this classification was intended to represent the extent of empirical anaerobic coverage rather than to imply clinical or pharmacological equivalence among the individual antimicrobial agents within each group. Although the agents within each category differ in their overall antimicrobial spectra and clinical indications, they were grouped according to the breadth of anaerobic activity relevant to the study question. Accordingly, patients were categorized as receiving LAC or EAC based on the anaerobic spectrum of the initial antimicrobial regimen administered on hospital day 1 and continued without change for at least 3 consecutive days. The LAC group comprised patients treated with ceftriaxone, cefotaxime, cefepime, cefozopran, or levofloxacin, which have activity against some oral anaerobes but limited or unreliable activity against a broad range of anaerobic Gram-negative bacilli, particularly the *Bacteroides fragilis* group. In contrast, the EAC group comprised patients treated with ampicillin–sulbactam, piperacillin–tazobactam, meropenem, doripenem, lascufloxacin, clindamycin, or metronidazole, which have broader activity against oral and gastrointestinal anaerobes. Lascufloxacin was classified as an EAC agent because in vitro antimicrobial activity against anaerobic bacteria, including oral anaerobes, has been reported [13]. Patients who received antimicrobial agents corresponding to both the LAC and EAC groups were classified into the EAC group.

The primary outcomes were 30-day in-hospital mortality and overall in-hospital mortality. The secondary outcome was length of hospital stay. As an additional outcome, we compared the duration of continued intravenous study antimicrobial therapy used to define either the LAC or EAC group.

### 2.4. Statistical Analysis

Statistical analyses were performed using R version 4.6.1 (R Foundation for Statistical Computing, Vienna, Austria). Continuous variables were compared using the Mann–Whitney U test, and categorical variables were compared using the chi-square test or Fisher’s exact test, as appropriate. Propensity score matching was performed using the MatchIt package (version 4.7.2). Covariate balance was assessed using standardized mean differences (SMDs) with the cobalt package (version 5.0.0). For inverse probability of treatment weighting (IPTW) analyses, weighted binary outcomes were analyzed using survey-weighted logistic regression, and weighted continuous outcomes were analyzed using survey-weighted linear regression with the survey package (version 4.5). A two-sided *p* value < 0.05 was considered statistically significant.

Propensity scores were estimated using a multivariable logistic regression model including prespecified baseline covariates considered clinically relevant to treatment selection and clinical outcomes: age, sex, body mass index (BMI), BMI missingness, Brinkman Index, Barthel Index, impaired consciousness, Charlson Comorbidity Index, Hugh–Jones classification, oxygen therapy, mechanical ventilation, and catecholamine use. Covariates were selected a priori based on clinical relevance rather than statistical significance of between-group differences.

Missing BMI values were imputed using the median, with a separate indicator variable for BMI missingness. Missing categories were used for the Brinkman Index, Barthel Index, and Hugh–Jones classification.

For the primary analysis, 1:1 propensity score matching without replacement was performed using Mahalanobis distance based on the covariates included in the propensity score model, restricted to pairs within a caliper of 0.2 standard deviations of the logit of the propensity score. Covariate balance before and after matching was assessed using absolute SMDs, with an absolute SMD < 0.1 considered indicative of adequate balance. Propensity score distributions before and after matching were visually assessed to evaluate overlap between the treatment groups.

Given the marked imbalance in treatment-group sizes, IPTW was additionally performed as a sensitivity analysis using stabilized weights targeting the average treatment effect. Covariate balance after weighting was assessed using absolute SMDs. To further assess potential selection bias associated with requiring continuation of the initial regimen for at least 3 consecutive days, baseline characteristics were compared between patients included in the primary analysis and those excluded because of an early regimen change. Differences were quantified using absolute SMDs. We also conducted an analysis in which exposure was defined according to the antimicrobial regimen administered on hospital day 1, irrespective of subsequent treatment modification. Because in-hospital death may truncate the length of hospital stay, length of stay was additionally evaluated among patients who survived to hospital discharge.

## 3. Results

### 3.1. Patient Characteristics

Among 3639 patients with ICD-10 code J85.2 recorded as the diagnosis prompting admission, 111 patients with second or subsequent hospitalizations during the study period, 454 patients who did not receive any study antimicrobial agent on hospital day 1, and 13 patients who died or were discharged on hospital day 1 were excluded. The remaining 3061 patients were included in the sensitivity analysis based on antimicrobial exposure on hospital day 1. Of these, 261 patients whose initial antimicrobial regimen was not continued for at least 3 consecutive days were excluded from the primary analysis, resulting in a primary analysis population of 2800 patients (Figure 1). Compared with patients included in the primary analysis, those excluded because of an early regimen change had lower functional status, more frequently had impaired consciousness, and more often required oxygen therapy, mechanical ventilation, and catecholamine support on hospital day 1; they were also less likely to have received EAC on hospital day 1 (Supplementary Table S1). Among the 2800 patients included in the primary analysis, 23.1% (648/2800) were female, and the median age was 74 years (interquartile range [IQR], 64–82 years). Based on the initial antimicrobial regimen administered on hospital day 1 and continued for at least 3 consecutive days, patients were classified into the LAC group (*n* = 161) and the EAC group (*n* = 2639).

In the LAC group, ceftriaxone was the most frequently used antimicrobial agent (132/161 patients, 82.0%), followed by levofloxacin (13/161, 8.1%), cefepime (11/161, 6.8%), cefotaxime (4/161, 2.5%), and cefozopran (2/161, 1.2%). In the EAC group, ampicillin–sulbactam was the most frequently used antimicrobial agent (1508/2639 patients, 57.1%), followed by piperacillin–tazobactam (791/2639, 30.0%), meropenem (258/2639, 9.8%), clindamycin (84/2639, 3.2%), lascufloxacin (15/2639, 0.6%), doripenem (12/2639, 0.5%), and metronidazole (8/2639, 0.3%). Because some patients received more than one study antimicrobial agent, the sum of the numbers of patients receiving individual antimicrobial agents exceeded the total number of patients in each group. Among the 2639 patients in the EAC group, 77 (2.9%) concomitantly received an LAC agent: ceftriaxone in 54 patients, cefepime in 11 patients, and levofloxacin in 12 patients, with no patient receiving more than one of these LAC agents.

Among the 2800 patients included in the primary analysis, 133 (4.8%) had a concomitant diagnosis of aspiration pneumonia. Aspiration pneumonia was recorded in 9 of 161 patients (5.6%) in the LAC group and 124 of 2639 patients (4.7%) in the EAC group.

### 3.2. Propensity Score Matching

Before matching, several baseline covariates showed between-group imbalance. Propensity scores were estimated using prespecified clinically relevant covariates, as described in the Methods. The propensity score model had an AUC of 0.616. After 1:1 propensity score matching, 161 patients were included in each group. Propensity score overlap improved after matching (Supplementary Figure S1), and covariate balance was assessed using absolute SMDs (Table 1), although some residual imbalance remained.

### 3.3. Comparison of Mortality and Length of Hospital Stay Between the LAC and EAC Groups

After propensity score matching, EAC therapy was not significantly associated with lower 30-day in-hospital mortality compared with LAC therapy (OR, 1.00; 95% CI, 0.36–2.76; *p* = 1.000). Similarly, no significant difference was observed in overall in-hospital mortality (OR, 0.83; 95% CI, 0.39–1.75; *p* = 0.600). Length of hospital stay also did not differ significantly between the two groups (*p* = 0.993). In a sensitivity analysis restricted to patients who survived to hospital discharge, the median length of hospital stay was 19 days (IQR, 14–30) in the LAC group and 20 days (IQR, 14–29) in the EAC group, with no significant between-group difference (*p* = 0.878) (Table 2).

The median duration of antimicrobial therapy was 13 days (IQR, 8–17 days) in the LAC group and 14 days (IQR, 11–19 days) in the EAC group and was shorter in the LAC group (*p* = 0.032).

### 3.4. Sensitivity Analyses

In the primary cohort of 2800 patients, IPTW improved the balance of measured baseline covariates, although some residual imbalance remained (Supplementary Table S2). After weighting, EAC therapy was not significantly associated with 30-day in-hospital mortality (OR, 1.09; 95% CI, 0.54–2.17; *p* = 0.815) or overall in-hospital mortality (OR, 0.78; 95% CI, 0.44–1.38; *p* = 0.399). No significant differences were observed in length of hospital stay or length of hospital stay among patients discharged alive (Supplementary Table S3).

To assess the potential influence of requiring continuation of the initial antimicrobial regimen for at least 3 consecutive days, we performed an additional sensitivity analysis in 3061 patients classified solely according to the antimicrobial regimen administered on hospital day 1, irrespective of subsequent treatment modification. After IPTW, there was no statistically significant association between EAC therapy and 30-day in-hospital mortality (OR, 0.76; 95% CI, 0.46–1.25; *p* = 0.274) or overall in-hospital mortality (OR, 0.80; 95% CI, 0.53–1.22; *p* = 0.299). Length of hospital stay also did not differ significantly between the groups (mean difference, −0.67 days; 95% CI, −3.54 to 2.21; *p* = 0.650) (Supplementary Tables S4 and S5).

## 4. Discussion

In this nationwide database study, more than 90% of patients received EAC therapy, indicating that extended anaerobic coverage is widely used for lung abscess in routine clinical practice in Japan. We found no statistically significant evidence that extended anaerobic coverage was associated with lower in-hospital mortality or a shorter length of hospital stay compared with limited anaerobic coverage. The findings were generally consistent across propensity score matching and IPTW analyses, including a sensitivity analysis in which exposure was defined solely according to the antimicrobial regimen administered on hospital day 1. However, the confidence intervals for the mortality estimates were relatively wide, and clinically important differences cannot be excluded. Therefore, these findings should be interpreted cautiously and should not be considered evidence of equivalence between the two treatment strategies.

Several explanations may account for the lack of significant differences in clinical outcomes between the two groups. First, causative pathogens of lung abscess are not limited to anaerobic bacteria; aerobic and facultative anaerobic bacteria, such as *Staphylococcus aureus* and members of the family Enterobacteriaceae, including *Klebsiella pneumoniae*, are also known to be involved in a certain proportion of cases [4,14]. Therefore, if the study population included patients in whom anaerobes were not the predominant pathogens, the additional therapeutic effect of anaerobic coverage may have been limited. Second, ceftriaxone, which accounted for the majority of antimicrobial therapy in the LAC group, may have activity against some oral anaerobic bacteria. Although ceftriaxone may have limited activity against beta-lactamase-producing anaerobes, including some *Prevotella* spp., it may retain activity against some oral anaerobes, including *Peptostreptococcus* spp. and *Fusobacterium* spp. [15,16]. Therefore, a certain degree of anaerobic treatment effect may have been achieved even in the LAC group, potentially reducing differences between the two groups.

Unnecessary antimicrobial exposure may increase selective pressure for antimicrobial resistance and contribute to antimicrobial-associated adverse events, including *Clostridioides difficile* infection (CDI) [3,17]. The risk of CDI varies among antimicrobial classes, and increased risks have been reported with several commonly used agents, including clindamycin, fluoroquinolones, and cephalosporins [18]. More recently, Bai et al. reported that extended anaerobic coverage did not improve mortality in patients with community-acquired aspiration pneumonia but was associated with a higher incidence of *C. difficile* colitis than limited anaerobic coverage [11]. However, aspiration pneumonia and lung abscess are clinically distinct entities, and findings from aspiration pneumonia should not be directly extrapolated to patients with lung abscess. Nevertheless, these findings highlight the potential for treatment-related harm associated with broader anaerobic coverage when no clear clinical benefit is obtained. In the present study, however, the number of CDI events was too small to permit a reliable comparison between the LAC and EAC groups. Therefore, differences in CDI risk could not be adequately evaluated, which should be considered when interpreting the present findings. Nevertheless, in the absence of a clear improvement in major clinical outcomes with extended anaerobic coverage, a more selective antimicrobial treatment strategy based on patient characteristics and disease severity may be preferable from the perspectives of AMR control and antimicrobial stewardship.

A strength of this study is that it used a nationwide DPC database, enabling a large-scale analysis of patients with lung abscess that would be difficult to achieve in a single-center study. In addition, propensity score matching was performed to adjust for baseline characteristics and reduce the influence of measured confounding in this observational study [19]. This study also has several limitations. First, the diagnosis of lung abscess was based on ICD-10 code J85.2 and clinical diagnoses recorded at each participating institution. Although a previous validation study of the Japanese DPC database demonstrated generally high specificity of diagnostic coding, sensitivity varied across diagnoses [9], and disease-specific validation data for ICD-10 code J85.2 are not available. Therefore, diagnostic misclassification and heterogeneity of the included cases cannot be excluded. Second, the DPC database does not include detailed clinical information such as imaging findings, inflammatory markers, abscess size, concomitant empyema, microbiological test results, or aspiration risk. These factors may influence both antimicrobial selection and clinical outcomes. Aspiration pneumonia may also have been under-recorded because it was identified only when documented as a concomitant diagnosis in the administrative database. In addition, the small number of patients with recorded aspiration pneumonia, particularly in the LAC group, precluded a reliable sensitivity analysis restricted to this subgroup. Clinicians may have preferentially selected EAC therapy for patients perceived to have more severe disease or a higher likelihood of infection with anaerobic or resistant pathogens. Although only a small proportion of patients in the EAC group received concomitant LAC agents (77/2639, 2.9%), residual bias related to combination therapy cannot be excluded. The antimicrobial agents within each group also differed in their overall antimicrobial spectra and clinical indications, which may have introduced within-group heterogeneity. Therefore, residual confounding by indication may remain despite propensity score adjustment. Third, the primary analysis required continuation of the initial antimicrobial regimen for at least 3 consecutive days, which may have introduced selection or conditioning bias by excluding patients whose treatment was changed early because of clinical deterioration, treatment failure, adverse events, or rapid improvement. The excluded patients differed clinically from those retained in the primary analysis, supporting the possibility of such bias. To address this concern, we performed a sensitivity analysis in which exposure was defined solely according to the antimicrobial regimen administered on hospital day 1, irrespective of subsequent treatment modification. The results were broadly consistent with those of the primary analysis. Fourth, treatment decisions may have been influenced by hospital-level prescribing practices or institutional antimicrobial policies. Because hospital-level clustering was not explicitly accounted for in the present analysis, residual confounding related to institutional prescribing practices may remain. Fifth, this study identified patients with lung abscess using ICD-10 code J85.2; therefore, patients coded as J85.1 (lung abscess with pneumonia) were not included. This approach may have preferentially identified patients in whom lung abscess was the predominant clinical condition while excluding some patients with lung abscess accompanied by pneumonia who were coded as J85.1. Consequently, the study population may represent a narrower subset of patients with lung abscess, which could have affected case ascertainment and the distribution of clinical characteristics. Therefore, caution is required when generalizing our findings to patients with lung abscess coded as J85.1 or to the broader population of patients with lung abscess. Sixth, the matched sample size and limited number of mortality events resulted in relatively wide confidence intervals and limited statistical precision for detecting modest differences in mortality. Based on the matched sample size of 161 patients per group, only relatively large absolute differences in mortality could be detected with 80% power, indicating limited ability to detect smaller but potentially clinically relevant differences. Therefore, clinically important differences between the treatment strategies cannot be excluded, and the absence of statistically significant differences should not be interpreted as evidence of equivalence or non-inferiority.

## 5. Conclusions

We found no statistically significant evidence that extended anaerobic coverage was associated with improved clinical outcomes in the overall study population. However, the potential benefit of extended anaerobic coverage in selected patients remains uncertain. Future studies incorporating microbiological, imaging, and clinical severity data are needed to identify patients who may benefit from extended anaerobic coverage and to develop risk-stratification or prediction models to guide individualized antimicrobial selection.

## Figures and Tables

**Figure 1:**
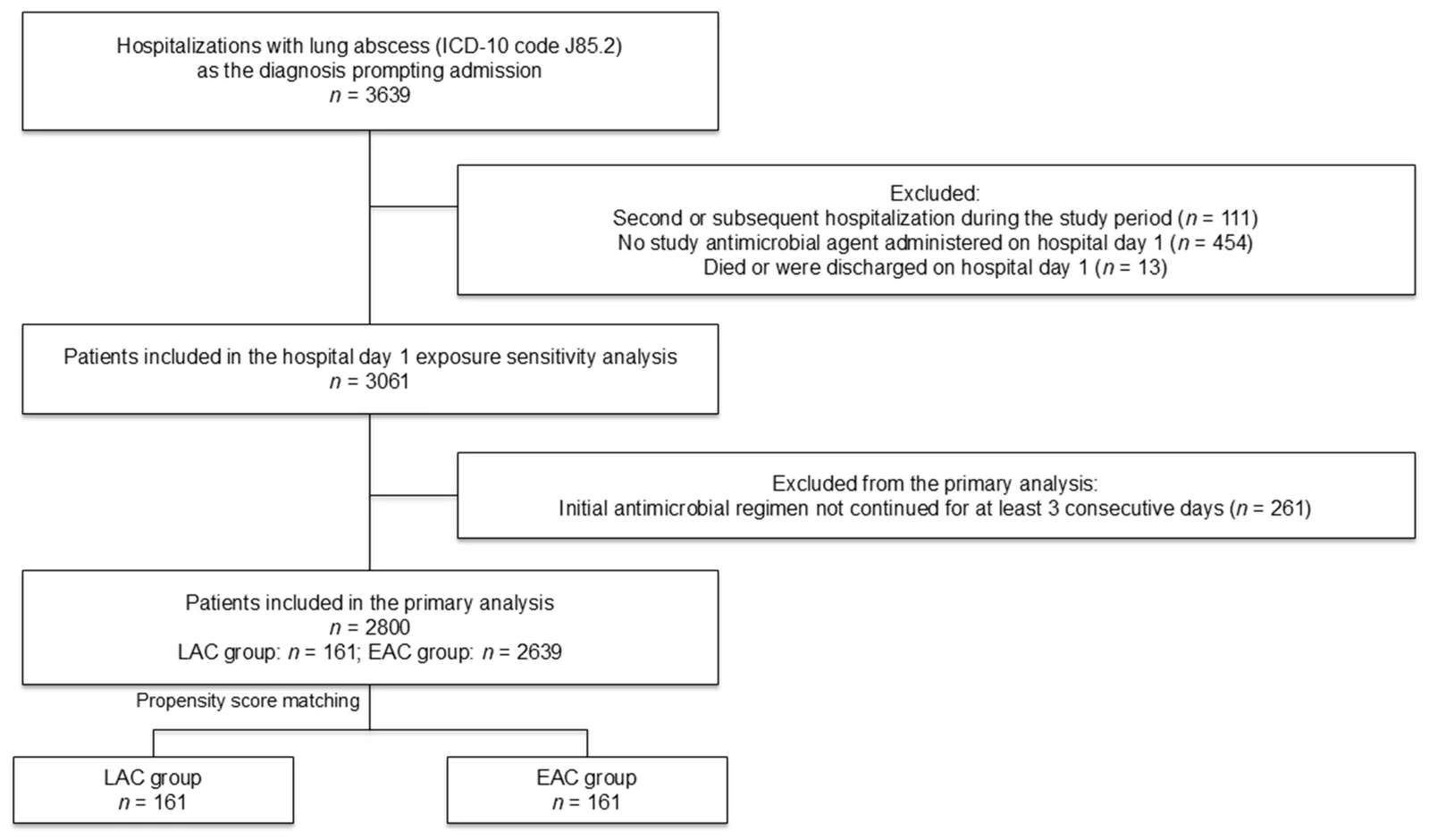
Flowchart of patient selection and propensity score matching.

**Table 1:** Covariate balance before and after propensity score matching in the primary cohort.

	Before Matching			After Matching		
Variable	LAC Group (*n* = 161)	EAC Group (*n* = 2639)	Absolute SMD	LAC Group (*n* = 161)	EAC Group (*n* = 161)	Absolute SMD
Age (years)	73.9 (14.3)	71.6 (13.8)	0.158	73.9 (14.3)	74.1 (13.9)	0.013
Sex (female)	36 (22.4%)	612 (23.2%)	0.020	36 (22.4%)	34 (21.1%)	0.030
BMI (kg/m^2^)	20.7 (3.8)	20.7 (4.0)	0.004	20.7 (3.8)	20.3 (3.5)	0.097
Missing	12 (7.5%)	201 (7.6%)	0.006	12 (7.5%)	7 (4.3%)	0.118
Brinkman Index						
0	64 (39.8%)	993 (37.6%)	0.043	64 (39.8%)	66 (41.0%)	0.025
1–399	18 (11.2%)	269 (10.2%)	0.031	18 (11.2%)	16 (9.9%)	0.039
400–799	21 (13.0%)	439 (16.6%)	0.107	21 (13.0%)	21 (13.0%)	0.000
≥800	35 (21.7%)	591 (22.4%)	0.016	35 (21.7%)	35 (21.7%)	0.000
Missing	23 (14.3%)	347 (13.1%)	0.032	23 (14.3%)	23 (14.3%)	0.000
Barthel Index						
0–55	43 (26.7%)	762 (28.9%)	0.049	43 (26.7%)	41 (25.5%)	0.028
60–95	32 (19.9%)	350 (13.3%)	0.166	32 (19.9%)	31 (19.3%)	0.016
100	67 (41.6%)	1308 (49.6%)	0.161	67 (41.6%)	70 (43.5%)	0.038
Missing	19 (11.8%)	219 (8.3%)	0.109	19 (11.8%)	19 (11.8%)	0.000
Impaired consciousness (JCS 1–300)	19 (11.8%)	399 (15.1%)	0.103	19 (11.8%)	19 (11.8%)	0.000
Charlson Comorbidity Index						
0	66 (41.0%)	1224 (46.4%)	0.110	66 (41.0%)	68 (42.2%)	0.025
1–2	70 (43.5%)	1048 (39.7%)	0.076	70 (43.5%)	74 (46.0%)	0.050
≥3	25 (15.5%)	367 (13.9%)	0.045	25 (15.5%)	19 (11.8%)	0.103
Hugh–Jones classification						
0	40 (24.8%)	538 (20.4%)	0.103	40 (24.8%)	39 (24.2%)	0.014
1	44 (27.3%)	731 (27.7%)	0.008	44 (27.3%)	45 (28.0%)	0.014
2	22 (13.7%)	447 (16.9%)	0.095	22 (13.7%)	20 (12.4%)	0.036
3	13 (8.1%)	296 (11.2%)	0.115	13 (8.1%)	13 (8.1%)	0.000
4	20 (12.4%)	369 (14.0%)	0.047	20 (12.4%)	21 (13.0%)	0.019
5	16 (9.9%)	222 (8.4%)	0.051	16 (9.9%)	17 (10.6%)	0.021
Missing	6 (3.7%)	36 (1.4%)	0.125	6 (3.7%)	6 (3.7%)	0.000
Comorbidities						
Respiratory failure	17 (10.6%)	270 (10.2%)	0.011	17 (10.6%)	13 (8.1%)	0.081
Heart failure	8 (5.0%)	143 (5.4%)	0.021	8 (5.0%)	7 (4.3%)	0.029
Chronic kidney disease	2 (1.2%)	60 (2.3%)	0.093	2 (1.2%)	3 (1.9%)	0.056
Asthma	5 (3.1%)	91 (3.4%)	0.020	5 (3.1%)	4 (2.5%)	0.036
COPD	9 (5.6%)	153 (5.8%)	0.009	9 (5.6%)	17 (10.6%)	0.216
Hypertension	32 (19.9%)	550 (20.8%)	0.024	32 (19.9%)	35 (21.7%)	0.047
Diabetes mellitus	33 (20.5%)	541 (20.5%)	0.000	33 (20.5%)	31 (19.3%)	0.031
Cancer	24 (14.9%)	409 (15.5%)	0.017	24 (14.9%)	26 (16.1%)	0.035
Interventions on day 1						
Oxygen therapy	46 (28.6%)	728 (27.6%)	0.022	46 (28.6%)	39 (24.2%)	0.096
Mechanical ventilation	1 (0.6%)	28 (1.1%)	0.056	1 (0.6%)	1 (0.6%)	0.000

Data are presented as *n* (%) for categorical variables and means (standard deviations) for continuous variables. Percentages and continuous variables are reported to one decimal place, and absolute SMDs to three decimal places. Absolute standardized mean differences (SMDs) were used to assess covariate balance, with an absolute SMD < 0.1 considered indicative of adequate balance. Propensity scores were estimated using logistic regression. Patients were matched 1:1 without replacement using Mahalanobis distance based on all covariates included in the propensity score model, within a caliper of 0.2 standard deviations of the logit of the propensity score. Missing BMI values were imputed using the median, with a separate missingness indicator included in the propensity score model. LAC, limited anaerobic coverage; EAC, extended anaerobic coverage; PSM, propensity score matching; SMD, standardized mean difference; BMI, body mass index; JCS, Japan Coma Scale; COPD, chronic obstructive pulmonary disease; ICU, intensive care unit.

**Table 2:** Clinical outcomes after propensity score matching in the primary cohort.

Outcome	After Matching		Effect Estimate (95% CI)	*p* Value
	LAC Group (*n* = 161)	EAC Group (*n* = 161)		
30-day in-hospital mortality	10 (6.2%)	10 (6.2%)	OR 1.00 (0.36–2.76)	1.000
Overall in-hospital mortality	20 (12.4%)	17 (10.6%)	OR 0.83 (0.39–1.75)	0.600
Length of hospital stay, days	20 (14–33)	20 (14–30)	—	0.993
Length of hospital stay among patients discharged alive, days	19 (14–30)	20 (14–29)	—	0.878

Data are presented as *n* (%) for mortality outcomes and medians (interquartile ranges) for continuous outcomes. Odds ratios (ORs) and 95% confidence intervals (CIs) are presented for mortality outcomes. *p* values for mortality outcomes were calculated using Pearson’s chi-square test, and length of hospital stay was compared using the Mann–Whitney U test. Odds ratios are presented for EAC therapy relative to LAC therapy. LAC, limited anaerobic coverage; EAC, extended anaerobic coverage; PSM, propensity score matching; OR, odds ratio; CI, confidence interval.

## Data Availability

The data used in this study are not publicly available due to restrictions on the use of the DPC database.

## Supplementary Materials

The following supporting information can be downloaded at https://www.mdpi.com/article/10.3390/pathogens15101013/s1, Supplementary Figure S1. Propensity score distributions before and after matching: (A) Propensity score distributions in the LAC and EAC groups before propensity score matching. (B) Propensity score distributions in the LAC and EAC groups after propensity score matching.; Table S1: Comparison of baseline characteristics between patients included in the primary cohort and those excluded because the initial antimicrobial regimen was not continued for at least 3 consecutive days.; Table S2: Covariate balance before and after inverse probability of treatment weighting in the primary cohort.; Table S3: Clinical outcomes after inverse probability of treatment weighting in the primary cohort.; Table S4: Covariate balance before and after inverse probability of treatment weighting in the day-1 exposure sensitivity analysis (*n* = 3061).; Table S5: Clinical outcomes after inverse probability of treatment weighting in the day-1 exposure sensitivity analysis (*n* = 3061).

## Abbreviations

AMR	antimicrobial resistance
AUC	area under the curve
BMI	body mass index
CCI	Charlson Comorbidity Index
CDI	*Clostridioides difficile* infection
CI	confidence interval
COPD	chronic obstructive pulmonary disease
DPC	Diagnosis Procedure Combination
EAC	extended anaerobic coverage
ICD-10	International Classification of Diseases, 10th Revision
ICU	intensive care unit
IPTW	inverse probability of treatment weighting
IQR	interquartile range
JCS	Japan Coma Scale
LAC	limited anaerobic coverage
MD	mean difference
OR	odds ratio
PSM	propensity score matching
SMD	standardized mean difference
