# Supplementary material for: Association Between Anaerobic Coverage and Clinical Outcomes in Patients with Lung Abscess: A Nationwide Database Study in Japan
Source: Pathogens. 2026 Sep 24;15(10):1013. doi: 10.3390/pathogens15101013 (PMC13708142; doi:10.3390/pathogens15101013)
Supplement: Supplementary file 1 [file pathogens-15-01013-s001.zip › 20260924_Matsumoto_Supplementary_Tables_S1-S5.pdf]

Supplementary Table S1. Comparison of baseline characteristics between patients included in the primary cohort and those excluded because the initial antimicrobial regimen was not continued for at least 3 consecutive days.

Variable	Included in primary analysis (n = 2800)	Initial regimen not continued for ≥3 consecutive days (n = 261)	Absolute SMD
Age (years)	74 (64–82)	74 (66–81)	0.037
Sex (female)	648 (23.1%)	69 (26.4%)	0.076
BMI (kg/m²)	20.4 (17.7–23.1)	20.5 (17.8–23.0)	0.012
Barthel Index			
0–55	805 (28.7%)	95 (36.4%)	0.164
60–95	382 (13.6%)	32 (12.3%)	0.041
100	1375 (49.1%)	95 (36.4%)	0.259
Missing	238 (8.5%)	39 (14.9%)	0.201
Impaired consciousness (JCS 1–300)	418 (14.9%)	71 (27.2%)	0.304
Charlson Comorbidity Index			
0	1290 (46.1%)	127 (48.7%)	0.052
1–2	1118 (39.9%)	97 (37.2%)	0.057
≥3	392 (14.0%)	37 (14.2%)	0.005
Hugh-Jones classification			
0	578 (20.6%)	66 (25.3%)	0.111
1	775 (27.7%)	54 (20.7%)	0.164
2	469 (16.8%)	40 (15.3%)	0.039
3	309 (11.0%)	28 (10.7%)	0.010
4	389 (13.9%)	27 (10.3%)	0.109
5	238 (8.5%)	38 (14.6%)	0.191
Missing	42 (1.5%)	8 (3.1%)	0.105
Interventions on day 1			
Oxygen therapy	774 (27.6%)	109 (41.8%)	0.300
Mechanical ventilation	29 (1.0%)	11 (4.2%)	0.200
Catecholamine use	45 (1.6%)	10 (3.8%)	0.137
ICU admission	18 (0.6%)	4 (1.5%)	0.086
Initial EAC therapy on hospital day 1	2639 (94.2%)	202 (77.4%)	0.498

Data are presented as median (interquartile range) for continuous variables and n (%) for categorical variables.
Absolute standardized mean differences (SMDs) were used to quantify differences in baseline characteristics, with an absolute SMD ≥0.1 considered indicative of a meaningful difference.
Patients included in the primary cohort received the same LAC or EAC regimen for at least 3 consecutive days, whereas patients excluded from the primary cohort did not continue the initial LAC or EAC regimen for at least 3 consecutive days.
Initial EAC therapy was defined according to the intravenous antimicrobial regimen administered on hospital day 1.
LAC, limited anaerobic coverage; EAC, extended anaerobic coverage; SMD, standardized mean difference; BMI, body mass index; JCS, Japan Coma Scale; ICU, intensive care unit.

Supplementary Table S2. Covariate balance before and after inverse probability of treatment weighting in the primary cohort.

	Before IPTW			After IPTW		
Variable	LAC group (*n* = 161)	EAC group (*n* = 2639)	Absolute SMD	LAC group (weighted)	EAC group (weighted)	Absolute SMD
Age (years)	73.9	71.6	0.161	70.9	71.7	0.058
Sex (female)	22.4%	23.2%	0.020	23.2%	23.1%	0.002
BMI (kg/m²)	20.7	20.7	0.004	20.7	20.7	0.008
Missing	7.5%	7.6%	0.006	6.5%	7.6%	0.040
Brinkman Index						
0	39.8%	37.6%	0.044	39.0%	37.7%	0.027
1–399	11.2%	10.2%	0.032	10.9%	10.3%	0.022
400–799	13.0%	16.6%	0.101	14.6%	16.4%	0.052
≥800	21.7%	22.4%	0.016	22.2%	22.4%	0.005
Missing	14.3%	13.1%	0.033	13.3%	13.2%	0.002
Barthel Index						
0–55	26.7%	28.9%	0.048	27.7%	28.7%	0.024
60–95	19.9%	13.3%	0.179	14.0%	13.6%	0.008
100	41.6%	49.6%	0.160	49.6%	49.1%	0.010
Missing	11.8%	8.3%	0.117	8.7%	8.5%	0.008
Impaired consciousness (JCS 1–300)	11.8%	15.1%	0.097	15.9%	14.9%	0.028
Charlson Comorbidity Index						
0	41.0%	46.4%	0.109	45.9%	46.1%	0.003
1–2	43.5%	39.7%	0.076	40.3%	39.9%	0.008
≥3	15.5%	13.9%	0.046	13.7%	14.0%	0.008
Hugh-Jones classification						
0	24.8%	20.4%	0.107	17.9%	20.6%	0.068
1	27.3%	27.7%	0.008	27.9%	27.7%	0.005
2	13.7%	16.9%	0.091	18.9%	16.8%	0.061
3	8.1%	11.2%	0.107	11.0%	11.0%	0.002
4	12.4%	14.0%	0.046	13.2%	13.9%	0.021
5	9.9%	8.4%	0.053	9.5%	8.5%	0.033
Missing	3.7%	1.4%	0.150	1.6%	1.5%	0.006

Comorbidities						
Respiratory failure	10.6%	10.2%	0.011	10.8%	10.2%	0.019
Heart failure	5.0%	5.4%	0.020	4.9%	5.4%	0.025
Chronic kidney disease	1.2%	2.3%	0.079	1.7%	2.3%	0.047
Asthma	3.1%	3.4%	0.019	2.6%	3.4%	0.046
COPD	5.6%	5.8%	0.009	5.3%	5.8%	0.021
Hypertension	19.9%	20.8%	0.024	17.7%	20.9%	0.078
Diabetes mellitus	20.5%	20.5%	0.000	20.2%	20.5%	0.008
Cancer	14.9%	15.5%	0.016	16.1%	15.5%	0.017
Interventions on day 1						
Oxygen therapy	28.6%	27.6%	0.022	24.2%	27.6%	0.076
Mechanical ventilation	0.6%	1.1%	0.048	1.9%	1.0%	0.098
Catecholamine use	0.6%	1.7%	0.098	1.9%	1.6%	0.030
Corticosteroid use	6.8%	5.6%	0.052	7.5%	5.6%	0.079
Immunosuppressant use	1.9%	1.0%	0.078	2.2%	1.0%	0.104
ICU admission	0.6%	0.6%	0.003	0.8%	0.6%	0.019

Data are presented as percentages for categorical variables and means for continuous variables. Percentages and continuous variables are reported to one decimal place, and absolute SMDs to three decimal places. Values after IPTW are weighted estimates.
Absolute standardized mean differences (SMDs) were used to assess covariate balance, with an absolute SMD <0.1 considered indicative of adequate balance.
The propensity score model included age, sex, BMI, BMI missingness, Brinkman Index, Barthel Index, impaired consciousness, Charlson Comorbidity Index, Hugh-Jones classification, oxygen therapy, mechanical ventilation, and catecholamine use. Missing BMI values were imputed using the median, with a separate missingness indicator included in the propensity score model.
LAC, limited anaerobic coverage; EAC, extended anaerobic coverage; IPTW, inverse probability of treatment weighting; SMD, standardized mean difference; BMI, body mass index; JCS, Japan Coma Scale; COPD, chronic obstructive pulmonary disease; ICU, intensive care unit.

Supplementary Table S3. Clinical outcomes after inverse probability of treatment weighting in the primary cohort.

Outcome	After IPTW (weighted)		Effect estimate (95% CI)	p value
	LAC group	EAC group		
30-day in-hospital mortality	4.6%	5.0%	OR 1.09 (0.54–2.17)	0.815
Overall in-hospital mortality	10.6%	8.5%	OR 0.78 (0.44–1.38)	0.399
Length of hospital stay, days	20 (13–34)	20 (14–31)	MD −2.10 (−5.97 to 1.77)	0.286
Length of hospital stay among patients discharged alive, days	20 (13–31)	20 (14–30)	MD 0.02 (−3.18 to 3.22)	0.989

Data are presented as weighted percentages for mortality outcomes and weighted medians (interquartile ranges) for continuous outcomes. Odds ratios (ORs) for mortality outcomes were estimated using weighted logistic regression models. Mean differences (MDs) for length of hospital stay were estimated using weighted linear regression models. Effect estimates are for EAC therapy relative to LAC therapy. Stabilized inverse probability of treatment weights targeting the average treatment effect were used. LAC, limited anaerobic coverage; EAC, extended anaerobic coverage; IPTW, inverse probability of treatment weighting; OR, odds ratio; MD, mean difference; CI, confidence interval.

Supplementary Table S4. Covariate balance before and after inverse probability of treatment weighting in the day-1 exposure sensitivity analysis (n = 3061).

	Before IPTW			After IPTW		
Variable	LAC group (n = 220)	EAC group (n = 2841)	Absolute SMD	LAC group (weighted)	EAC group (weighted)	Absolute SMD
Age (years)	74.5	71.5	0.220	70.9	71.7	0.056
Sex (female)	23.6%	23.4%	0.005	24.5%	23.4%	0.024
BMI (kg/m²)	20.7	20.7	0.006	20.6	20.7	0.026
Missing	10.5%	7.7%	0.096	7.2%	7.9%	0.023
Brinkman Index						
0	41.8%	37.9%	0.079	38.1%	38.2%	0.003
1–399	9.5%	10.3%	0.027	11.5%	10.3%	0.039
400–799	11.8%	16.2%	0.126	14.6%	15.9%	0.036
≥800	21.8%	22.2%	0.009	22.9%	22.2%	0.017
Missing	15.0%	13.3%	0.048	12.9%	13.5%	0.015
Barthel Index						
0–55	29.1%	29.4%	0.007	29.4%	29.4%	0.000
60–95	18.2%	13.2%	0.138	13.1%	13.5%	0.011
100	42.7%	48.4%	0.115	48.2%	48.0%	0.004
Missing	10.0%	9.0%	0.035	9.2%	9.1%	0.006
Impaired consciousness (JCS 1–300)	16.4%	15.9%	0.011	16.7%	16.0%	0.021
Charlson Comorbidity Index						
0	40.9%	46.7%	0.117	46.5%	46.3%	0.004
1–2	43.6%	39.4%	0.086	39.5%	39.7%	0.004
≥3	15.5%	13.9%	0.044	14.0%	14.0%	0.000
Hugh-Jones classification						
0	26.8%	20.6%	0.147	18.8%	21.0%	0.052
1	23.6%	27.3%	0.085	27.1%	27.1%	0.000
2	13.6%	16.9%	0.090	19.3%	16.6%	0.074
3	7.7%	11.3%	0.121	11.8%	11.0%	0.026
4	12.7%	13.7%	0.027	12.7%	13.6%	0.025
5	11.4%	8.8%	0.084	8.8%	9.0%	0.006
Missing	4.1%	1.4%	0.162	1.5%	1.6%	0.009

Comorbidities						
Respiratory failure	10.5%	10.7%	0.008	8.4%	10.8%	0.078
Heart failure	9.1%	5.3%	0.148	7.2%	5.3%	0.073
Chronic kidney disease	3.6%	2.6%	0.062	2.7%	2.6%	0.007
Asthma	5.5%	3.3%	0.107	5.8%	3.3%	0.123
COPD	5.0%	5.9%	0.039	4.2%	5.9%	0.077
Hypertension	24.1%	20.1%	0.095	22.0%	20.2%	0.044
Diabetes mellitus	23.6%	20.2%	0.084	23.4%	20.2%	0.077
Cancer	12.7%	15.7%	0.084	12.7%	15.8%	0.087
Interventions on day 1						
Oxygen therapy	31.4%	28.7%	0.059	26.3%	28.8%	0.055
Mechanical ventilation	1.8%	1.3%	0.045	1.3%	1.3%	0.003
Catecholamine use	1.4%	1.8%	0.037	2.2%	1.8%	0.035
Corticosteroid use	6.4%	5.7%	0.026	7.0%	5.8%	0.053
Immunosuppressant use	1.4%	0.9%	0.042	1.7%	0.9%	0.070
ICU admission	0.5%	0.7%	0.037	0.7%	0.7%	0.002

Data are presented as percentages for categorical variables and means for continuous variables. Percentages and continuous variables are reported to one decimal place, and absolute SMDs to three decimal places. Values after IPTW are weighted estimates. Exposure was defined according to the initial intravenous antibiotic regimen administered on hospital day 1.

Absolute standardized mean differences (SMDs) were used to assess covariate balance, with an absolute SMD <0.1 considered indicative of adequate balance.

The propensity score model included age, sex, BMI, BMI missingness, Brinkman Index, Barthel Index, impaired consciousness, Charlson Comorbidity Index, Hugh-Jones classification, oxygen therapy, mechanical ventilation, and catecholamine use. Missing BMI values were imputed using the median, with a separate missingness indicator included in the propensity score model.

LAC, limited anaerobic coverage; EAC, extended anaerobic coverage; IPTW, inverse probability of treatment weighting; SMD, standardized mean difference; BMI, body mass index; JCS, Japan Coma Scale; COPD, chronic obstructive pulmonary disease; ICU, intensive care unit.

Supplementary Table S5. Clinical outcomes after inverse probability of treatment weighting in the day-1 exposure sensitivity analysis (*n* = 3061).

Outcome	After IPTW (weighted)		Effect estimate (95% CI)	p value
	LAC group	EAC group		
30-day in-hospital mortality	7.5%	5.8%	OR 0.76 (0.46–1.25)	0.274
Overall in-hospital mortality	11.2%	9.2%	OR 0.80 (0.53–1.22)	0.299
Length of hospital stay, days	21 (13–32)	20 (14–31)	MD −0.67 (−3.54 to 2.21)	0.650
Length of hospital stay among patients discharged alive, days	21 (14–32)	20 (14–30)	MD 0.07 (−2.60 to 2.74)	0.960

Data are presented as weighted percentages for mortality outcomes and weighted medians (interquartile ranges) for continuous outcomes. Odds ratios (ORs) for mortality outcomes were estimated using weighted logistic regression models. Mean differences (MDs) for length of hospital stay were estimated using weighted linear regression models. Effect estimates are for EAC therapy relative to LAC therapy. Stabilized inverse probability of treatment weights targeting the average treatment effect were used. Exposure was defined according to the initial intravenous antibiotic regimen administered on hospital day 1. LAC, limited anaerobic coverage; EAC, extended anaerobic coverage; IPTW, inverse probability of treatment weighting; OR, odds ratio; MD, mean difference; CI, confidence interval.
